# Association between CYP1A2 gene variants −163 C/A (rs762551) and −3860 G/A (rs2069514) and bladder cancer susceptibility

**DOI:** 10.1186/s12885-024-12553-7

**Published:** 2024-07-22

**Authors:** Muhammad Sarfaraz Iqbal, Nimra Sardar, Kaoqing Peng, Layla A. Almutairi, Xialo Duan, Fouzia Tanvir, Kotb A. Attia, Gouhua Zeng, Di Gu

**Affiliations:** 1 Department of Urology, Minimally Invasive Surgery Center, Guangdong Key Laboratory of Urology, Guangzhou Urology Research Institute, The First Affiliated Hospital of Guangzhou Medical University, Guangzhou, China; 2 Department of Microbiology and Molecular Genetics, School of Applied Sciences, University of Okara, Okara, Pakistan; 3 Department of Biology, College of Science, Princess Nourah bint Abdulrahman University, P.O. Box 84428, Riyadh, 11671 Saudi Arabia; 4 Department of Zoology, Institute of Pure and Applied Zoology, University of Okara, Okara, Pakistan; 5 Center of Excellence in Biotechnology Research, King Saud University, P.O. Box 2455, Riyadh, 11451 Saudi Arabia

**Keywords:** CYP1A2-3860 G/A bladder cancer (BLCA), Muscle-invasive bladder cancer (MIBLCA), Non-muscle invasive bladder cancer (NMIBLCA)

## Abstract

**Background:**

Bladder cancer (BLCA) poses a significant global health challenge due to its high incidence, poor prognosis, and limited treatment options.

**Aims and objectives:**

This study aims to investigate the association between two specific polymorphisms, CYP1A2-163 C/A and CYP1A2-3860G/A, within the Cytochrome P450 1A2 (CYP1A2) gene and susceptibility to BLCA.

**Methods:**

The study employed a case-control design, genotyping 340 individuals using Polymerase Chain Reaction-High-Resolution Melting Curve (PCR-HRM). Various genetic models were applied to evaluate allele and genotype frequencies. Genetic linkage analysis was facilitated using R packages.

**Results:**

The study reveals a significant association with the − 163 C/A allele, particularly in the additive model. Odds ratio (OR) analysis links CYP1A2-163 C/A (rs762551) and CYP1A2-3860G/A(rs2069514) polymorphisms to BLCA susceptibility. The rs762551 C/A genotype is prevalent in 55% of BLCA cases and exhibits an OR of 2.21. The A/A genotype has an OR of 1.54. Regarding CYP1A2-3860G/A, the G/A genotype has an OR of 1.54, and the A/A genotype has an OR of 2.08. Haplotype analysis shows a predominant C-C haplotype at 38.2%, followed by a C-A haplotype at 54.7%, and a less frequent A-A haplotype at 7.1%. This study underscores associations between CYP1A2 gene variants, particularly rs762551 (CYP1A2-163 C/A), and an increased susceptibility to BLCA. Haplotype analysis of 340 individuals reveals a predominant C-C haplotype at 38.2%, followed by a C-A haplotype at 54.7%, and a less frequent A-A haplotype at 7.1%.

**Conclusion:**

In conclusion, the − 163 C/A allele, C/A genotype of rs762551, and G/A genotype of rs2069514 emerge as potential genetic markers associated with elevated BLCA risk.

**Supplementary Information:**

The online version contains supplementary material available at 10.1186/s12885-024-12553-7.

## Introduction

Bladder cancer (BLCA) represents a formidable challenge in the field of oncology, being a frequently occurring malignant disease with a high incidence and poor prognosis [1, 2], affecting approximately 549,000 new cases and resulting in approximately 200,000 deaths annually worldwide [3]. Notably, the incidence of BLCA is observed to be higher in Western nations compared to Asian nations [2, 4–6]. BLCA is classified into two major types, namely non-muscle-invasive bladder cancer (NMIBLCA) and muscle-invasive bladder cancer (MIBLCA), with the latter being associated with a higher incidence of metastasis [7]. SNPs in regulatory regions or gene promoters have been demonstrated to change gene expression and increase the risk of bladder cancer [4, 8, 9]. In recent years, several low-penetrance genes have emerged as potential candidates for bladder cancer risk [6, 9]. A vital cytochrome P450 enzyme-encoding gene, cytochrome P4501A2 (CYP1A2) is involved in pre-carcinogen activation and the metabolism of a variety of therapeutic medicines. The CYP1A2 gene has seven exons and six introns and is situated on the 15q24.1 chromosomal region of DNA. Its length is roughly 7.8 kb. The liver is the primary site of expression for the 515-residue protein CYP1A2, which has a molecular mass of 58294Da. However, it has also been observed that the pancreas and lungs express this enzyme [7, 10]. An essential enzyme in the pathophysiology of bladder cancer, CYP1A2 is involved in the activation of the two main known bladder carcinogens, aromatic amines (AAs) and polycyclic aromatic hydrocarbons (PAHs) [11–14].

CYP1A2 is widely acknowledged for its pivotal role in the metabolism of various exogenous substances. It exhibits significant expression not only in the human liver but also in diverse tissues such as the duodenum, ovary, heart, kidney, and mammary gland [15–17]. Dysregulated CYP1A2 expression has been associated with the onset of various human cancers, including hepatocellular carcinoma, breast cancer, prostate cancer, bladder cancer, and endometrial tumors [13]. Single-nucleotide polymorphisms (SNPs) represent the most prevalent form of genetic variation and are emerging as valuable genetic biomarkers [9]. They possess the potential to influence gene regulation by altering gene sequences, subsequently affecting their functional attributes. An increasing number of SNPs have been linked to bladder cancer, and functional polymorphisms within the CYP1A2 gene have been identified as modulators of its activity, with implications for cancer susceptibility at various anatomical sites [9]. The CYP1A2 gene has been reported to house more than 40 single nucleotide polymorphisms, underscoring its potential role in the etiological risk of cancer. However, among the numerous SNPs identified in the CYP1A2 gene, not all have demonstrated a discernible impact on CYP1A2 activity [9, 17]. Of note, two frequently investigated CYP1A2 polymorphisms are rs762551 (A > C) and rs2069514 (G/A) [18]. Furthermore, it is imperative to consider other genomic variations that may contribute to inter-individual differences in CYP1A2 expression, underscoring the complexity of comprehending the role of CYP1A2 in cancer susceptibility [8, 19].

Numerous studies have reported a link between CYP1A2 polymorphisms and the risk of bladder cancer (BLCA) to date [15]. However, the presence of conflicting results concerning the association between the CYP1A2-163 C/A and CYP1A2-3860 G/A polymorphisms and the risk of bladder carcinoma can be attributed, in part, to limited sample sizes and the modest impact of genetic variations on the development of bladder cancer [16]. Notably, no prior study has explored the association between these polymorphisms and BLCA risk in Pakistani cohorts [20]. Consequently, the current study seeks to assess the relationship between the CYP1A2-163 C/A polymorphism and BLCA risk, as well as to comprehensively evaluate the potential associations of specific SNPs within the CYP1A2 gene with the risk of BLCA. By scrutinizing the connections between CYP1A2 polymorphisms and BLCA risk, this research endeavors to contribute to the existing understanding of the genetic determinants influencing susceptibility to bladder cancer, potentially informing the development of targeted strategies for prevention and treatment of BLCA.

To date, there has been a significant paucity of prior studies specifically investigating the potential correlation between variations in the CYP1A2 gene and susceptibility to bladder cancer. Therefore, the current study was initiated with the primary aim of examining the potential impact of genetic variants within the CYP1A2 gene, specifically rs762551 and rs2069514, on an individual’s vulnerability to bladder cancer. The results of the analysis demonstrate a significant elevation in CYP1A2 expression within bladder urothelial carcinoma when compared to normal tissue. This assertion is supported by data obtained from the following source: http://ualcan.path.uab.edu/cgi-bin/TCGAExResultNew2.pl?genenam=CYP2C8&ctype=BLCA. Based on the facts described above, it is reasonable to hypothesize that genetic changes in the CYP1A2 gene could potentially contribute to the development of bladder cancer.

## Materials and methods

### Study subjects

From January 2022 to March 2023, a comprehensive and thorough methodology was employed to ascertain and enlist a cohort of 170 persons who had been diagnosed with bladder cancer (BLCA) at Jinnah Hospital in Lahore, Pakistan. To ensure research group integrity and relevance, medical data was analyzed to select participants, and strict inclusion criteria were applied. The trial group included only patients who met strict criteria, including a certified BLCA diagnosis and ongoing, individualized treatment for their malignancies. It’s important to note that the study thoroughly excluded people with other cancers. A 170-person control group was carefully selected from healthy people who had freely attended normal general health exams at Jinnah Hospital Lahore. Control volunteers for this study were selected using rigorous criteria. As shown in Table 1, these criteria included being 60 years or older, not smoking or drinking, not having any chronic illnesses, no severe medical histories, and no history of cancer or bladder-related disorders. All study participants, including patients and controls, were ethnic Pakistani population. The research protocol completed a thorough process of obtaining ethical approval from the Ethics Committee of the University of Okara before the recruitment of volunteers for the study. The voluntary provision of signed consent strictly regulated every participant’s participation.

**Table 1:** Baseline characteristics of bladder cancer patients and control subjects

Characteristics	Patients	Control
Age (mean ± SD)	59.08 (± 8.10)	71.32 (± 8.59)
BMI (Kg/m^2^)(mean ± SD)	24.77(± 1.50)	26.71 (± 1.54)
Bladder volume (mean ± SD)	42.41 (± 15.96)	42.50(± 17.21)
BLCA level(mean ± SD)	16.04 (± 1.02)	17.18 (± 1.01)
Gleason_Score (mean ± SD)	8.12 (± 1.457)	
**Gleason_Score Frequency Percentage**		
6	31	18.2%
7	37	21.8%
8	40	23.6%
9	39	22.7%
10	23	13.6%
**Clinical Stage**		
Stage 1(n, %)	51, 25.5%	47, 23.5%
Sage 2(n, %)	52, 26.0%	60, 30.0%
Stage 3(n, %)	41, 20.5%	49, 24.5%
Stage 4(n, %)	56, 28.0%	44, 22.0%
Smoking (n, %)	92, 46.0%	81, 40.5%
Alcohol Consumption	40, 20.0%	45, 22.5%

### SNP selection criteria

First, the precise location of the gene, the CYP1A2 gene, was determined. The gene under consideration is located on chromosome 15 at precise coordinates 74,748,845 − 74,756,607 on the forward strand, after the assembly of GRCh38. The gene cytochrome P450 family 1 subfamily A member 2, often known as CP12 and P3-450, is denoted by the HGNC Symbol HGNC:2596.

To ensure the utmost levels of data quality, rigorous quality control standards were established utilizing Haploview software. The criteria employed in this study encompassed the establishment of a minimum frequency threshold for minor alleles surpassing 5%, the verification of the presence of the minor genotype in over 75% of the samples, the maintenance of a linkage disequilibrium measure (r^2) below 0.8, and the assessment of adherence to Hardy-Weinberg equilibrium (HWE) standards, as evidenced by a p-value exceeding 0.05. Following a comprehensive quality control protocol, a total of four distinct single nucleotide polymorphisms (SNPs) were selected for subsequent investigation in the study. The SNPs rs762551 and rs2069514 were explicitly incorporated into the analysis.

The methodology employed for the identification of these specific single nucleotide polymorphisms (SNPs) entailed determining the precise genomic position of the CYP1A2 gene on chromosome 10, which spans from 95,036,772 to 95,069,497. The study obtained the data from the human GRCh38 database, which can be accessible at http://asia.ensembl.org/Homo_sapiens/Info/Index. Verification of gene selection was performed using The Cancer Genome Atlas (TCGA) database to ensure relevance and accuracy.

### Genotyping

The informed written consent of all participants was obtained, and a volume of 3 mL of blood was collected into a vial that contained EDTA. After being collected, the whole blood was promptly stored in a refrigerator at -20 °C until it could be further processed. The phenol-chloroform procedure was employed to extract genomic DNA from the blood samples, which were then stored at a temperature of -20 °C for genotyping analysis. The phenol-chloroform procedure was utilized to isolate genomic DNA from the samples, and the resultant DNA was thereafter kept at a temperature of -20 °C for later genotyping analysis.

The genotyping analysis was performed using high-resolution melting (HRM) curve analysis on a Light Cycler 480 PCR machine. The samples were amplified in 20-L reactions with the Light Cycler 480 HRM-Master Kit from Roche Diagnostics in Vienna, Austria, and analyzed on an LC480 instrument I (Roche Diagnostics GmbH, Mannheim, Germany). The primer sequences for identifying the CYP1A2-163 C/A (rs762551) and CYP1A2–3860 G/A polymorphisms and the PCR cycling conditions for CYP1A2-163 C/A (rs762551) and CYP1A2–3860 G/A are detailed in Table 2.

**Table 2:** Primer sequences used for HRM-PCR

SNP (rs)	Primer Sequence (5’→3’)	Temperature	PCR conditions
-163 C/A (rs762551)	F: AATCTTGAGGCTCCTTTCCA R: AGCTGGATACCAGAAAGACTAAGC	58 ˚C	Initial denaturation 95˚C/5 min; 95˚C/10 sec,58˚C/10 sec, 72˚C/10 sec, 40 cycles; final extension 72˚C/4 min
-3860 G/A(rs2069514)	F: CTGTGAACATGTCCAGGCG R: CCTCAGAATGGTGGTGTCTT	56 ˚C	Initial denaturation 95˚C/5 min; 95˚C/10 sec,56˚C/10 sec, 72˚C/10 sec, 40 cycles; final extension 72˚C/4 min.

### Haplotype analysis

To explore potential associations between CYP1A2 gene polymorphisms (rs762551 and rs2069514) and the vulnerability to Bladder cancer (BLCA), a comprehensive analysis of haplotypes was conducted. Haplotype analysis was carried out utilizing three core R programs: SNPstat and haplostats, LD heatmap, and p-heatmap.

### Statistical analysis

Frequencies of Genotype for both BLCA patients and healthy controls were calculated. 𝜒2 test, Odds ratio (OR), 95% confidence interval (95% CI), and Fisher’s exact test were used to compare the Genotype distributions and associations with selected clinical data. The statistical significance level was set at 𝑝 ≤ 0.05. HW diagnostics software Version 1 beta was used to calculate and analyze genotype frequencies for Hardy Weinberg equilibrium (Fox Chase Cancer Center, Philadelphia, PA).

## Results

### Clinical and pathological features, and multivariate analysis

**Fig. 1:**
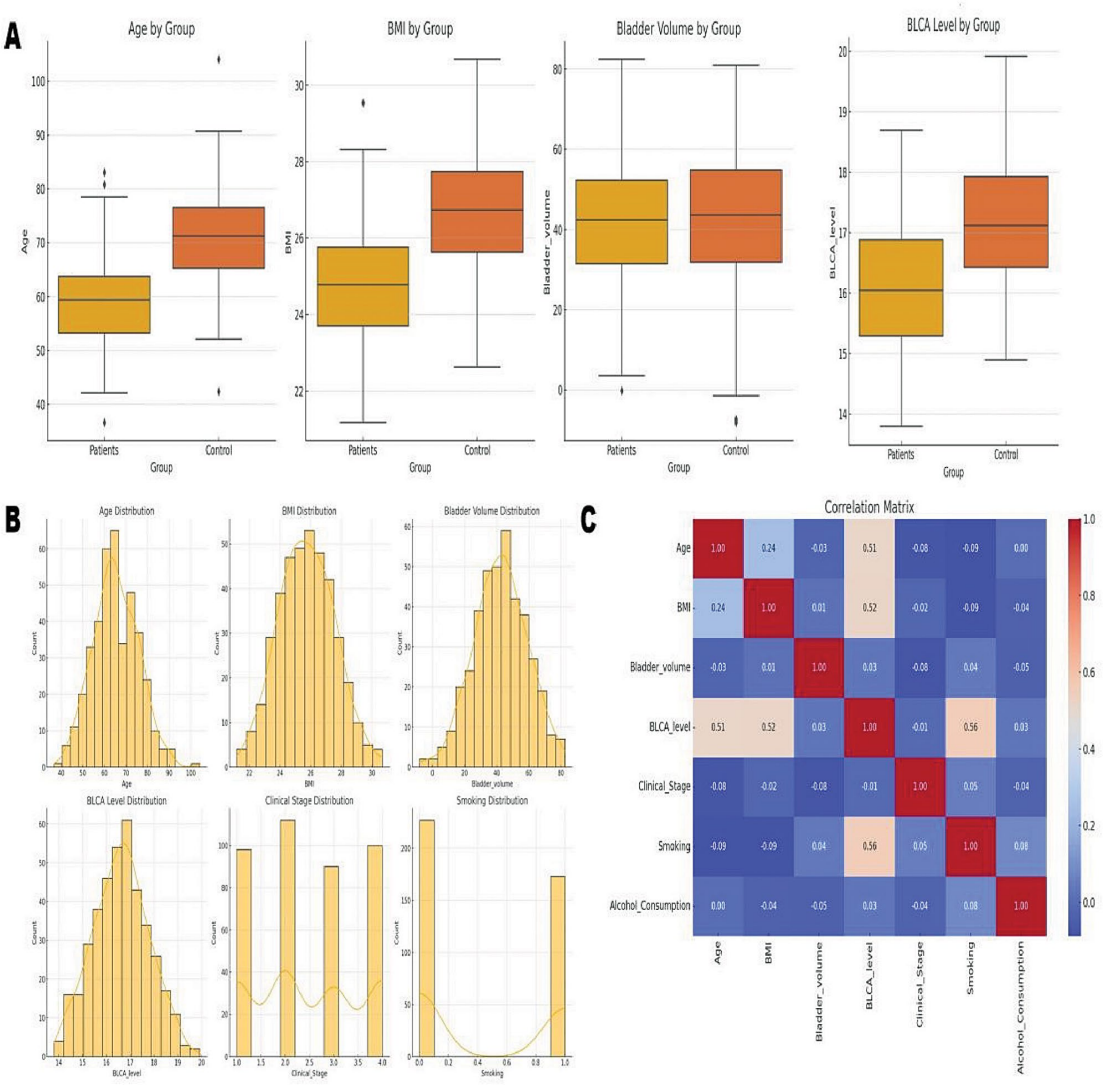
Multivariate Analysis: This figure presents a multivariate analysis of age, gender, smoking status, alcohol consumption, and CYP1A2 gene variants. Panels (**A**) and (**B**) show variable comparisons and distributions within the cohort. In contrast, panel (**C**) displays the coefficient matrix from the multivariate risk model, providing an adjusted assessment of the independent risk associated with genetic factors

The study explored the association between bladder cancer and specific variants in the CYP1A2 gene (-163 C/A and − 3860G/A) among 170 patients, including 102 with low-grade tumors and 68 with high-grade tumors. At the time of diagnosis, detailed records of age, gender, smoking habits, alcohol consumption, and BMI were collected for multivariate analysis. The results indicated that older age significantly increased the risk of bladder cancer, with a coefficient of 1.705649 (Fig. 1). Lower BMI was also correlated with higher cancer risk, evidenced by a coefficient of 1.229424. Smoking emerged as a significant risk factor, demonstrated by a coefficient of 0.771233, underscoring the importance of smoking cessation. Additionally, alcohol consumption was linked to a higher risk of bladder cancer, with a coefficient of 1.137675, suggesting the need for further investigation. The model’s intercept, at 0.24486270427169213, represented the baseline risk of bladder cancer when all other factors were held constant.

Higher expression levels of CYP1A2 were associated with improved survival rates, particularly in non-smokers and women with early-stage bladder cancer. Data from the TCGA revealed that CYP1A2 expression is significantly elevated in tumor tissues compared to normal tissues, highlighting its potential role in bladder cancer development and its promise as a therapeutic target (Figures S1 and S2). Using PCR-HRM, the genotypes for CYP1A2 gene SNPs were determined, as shown in Table 3. The varying levels of CYP1A2 expression during bladder cancer progression suggest it could serve as a valuable biomarker. Specifically, survival analysis showed that higher CYP1A2 expression is significantly associated with better survival rates in non-smokers and women with early-stage bladder cancer, supported by p-values of less than 0.05. Distinct trends were also observed about alcohol use and age, where CYP1A2 expression correlated with survival outcomes. The comparison between tumor and normal tissues revealed a significant elevation of CYP1A2 in tumor tissues, with p-values below 0.05, reaffirming its role in bladder cancer development. Furthermore, the analysis of smoking status indicated differing survival outcomes for smokers with higher CYP1A2 expression compared to non-smokers, with all p-values and standard deviations detailed in Figure S1.

**Table 3:** CYP1A2 -163 C/A and − 3860 G/A distribution in bladder cancer cases and controls

-163 C/A (rs762551)					-3860 G/A (rs2069514)				
Cases			Control		Cases			Control	
Genotype	Observed	HE expected	Observed	HE expected	Genotype	Observed	HE expected	Observed	HE expected
CC	65(38.24)	73.13(43.02)	102(60.00)	102.49(60.29)	GG	86(50.59.)	87.55(51.50)	109(64.12)	109.60(64.47)
CA	93(54.71)	76.74(45.14)	60(35.29)	59.01(34.71)	GA	72(42.35)	68.89(40.53)	55(32.35)	53.80(31.65)
AA	12(7.06)	20.13(11.88)	8(4.71)	8.49(5.00)	AA	12(7.06)	13.55(7.97)	6(3.53)	6.60(3.88)

### Allele frequency and genotype analysis

**Fig. 2:**
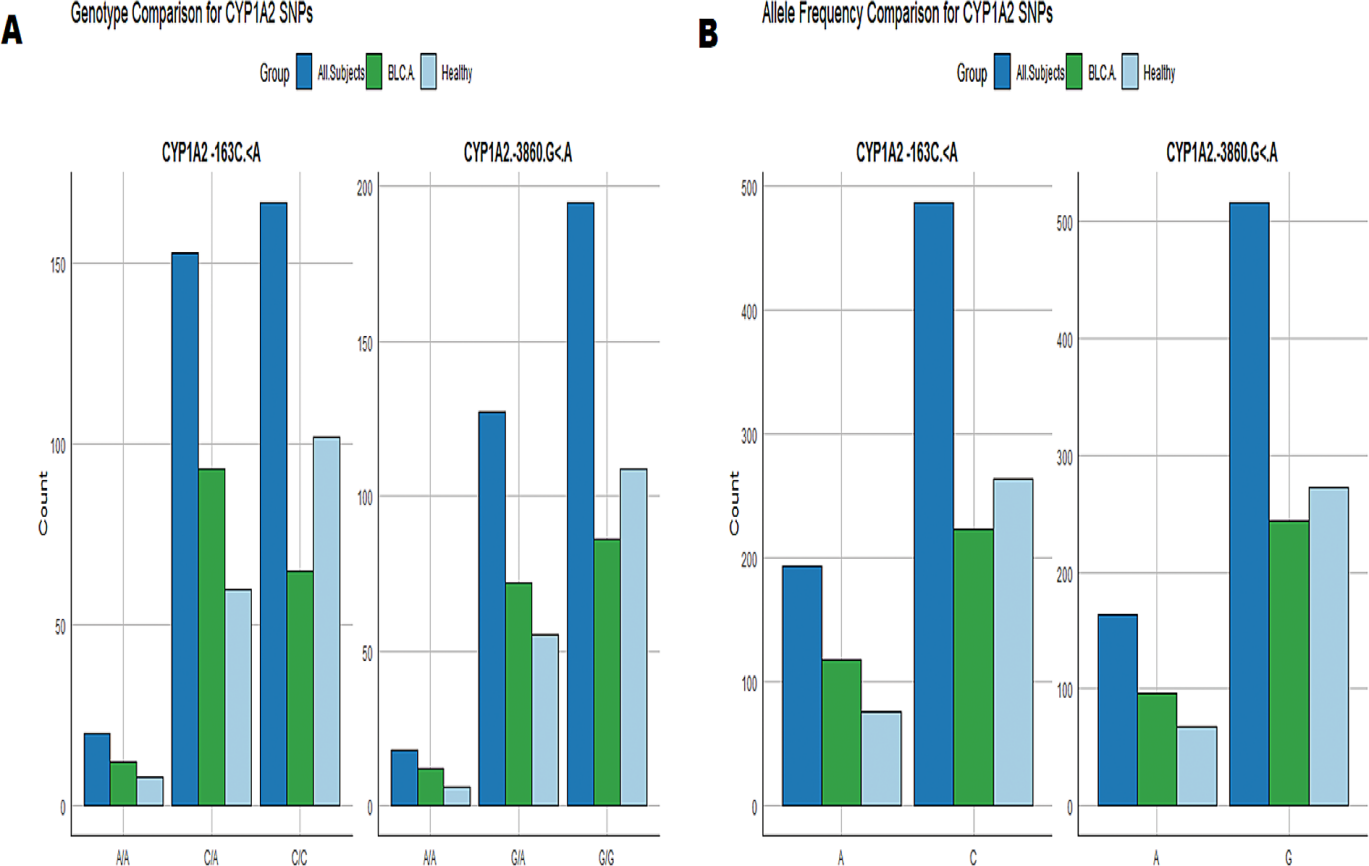
(**A**) The genotype comparison of SNPs CYP1A2-163 C.< A and CYP1A2-3860.G.< A, (**B**) The allele frequency comparison for SNPs CYP1A2-163 C.< A and CYP1A2-3860.G.< A

Upon examining the genotype distribution of rs762551 and rs2069514, a clear difference was observed between the control group and BLCA patients. Specifically, fewer individuals in the control group had the heterozygous CA genotype of rs762551 (Table 4). Moreover, a higher proportion of individuals in the control group had the AA genotype of rs2069514, while more BLCA patients had the GG genotype, as detailed in Table 4. The comparison of genotypes for SNPs CYP1A2-163 C/A and CYP1A2-3860G/A (Fig. 2A). These results suggest that these genetic variants may play a role in the development or progression of BLCA, warranting further investigation.Additionally, compared to the healthy control group, BLCA patients showed significantly lower frequencies of the C allele (0.66%) and the CC genotype (0.38%) in rs762551. Conversely, the “A” allele frequency (0.34%) and AA genotype frequency (0.07%) were higher in patients than in the control group, which had frequencies of 0.22% and 0.05%, respectively (Table 4; Fig. 2B).

**Table 4:** Genotype analysis between CYP1A2 rs762551 and rs2069514 SNPs

SNP	Genotype	All.Subjects (*n* = 340)	BLC.A.(*n* = 170)	Healthy (*n* = 170)
CYP1A2 -163 C.< A (rs762551)	C/C	167(0.49)	65(0.38)	102(0.6)
	C/A	153(0.45)	93(0.55)	60(0.35)
	A/A	20(0.06)	12(0.07)	8(0.05)
	C	487(0.72)	223(0.66)	264(0.78)
	A	193(0.28)	117(0.34)	76(0.22)
CYP1A2.-3860.G<.A (rs2069514)	G/G	195(0.57)	86(0.51)	109(0.64)
	G/A	127(0.37)	72(0.42)	55(0.32)
	A/A	18(0.05)	12(0.07)	6(0.04)
	G	517(0.76)	244(0.72)	273(0.8)
	A	163(0.24)	96(0.28)	67(0.2)

### Genotype and allele frequency distribution for CYP1A2-163 C < A

Three distinct genotypes (C/C, C/A, and A/A) were identified among both BLCA) cases and control subjects. The genotype distributions in both groups conformed to the Hardy-Weinberg equilibrium (HWE), thus affirming the reliability of the genotyping data (Table 5). Among these genotypes, the − 163 C/A CA genotype (X² = 12.941, OR = 2.21; 95% CI = 1.43–3.42; *P* = 0.000321) and the A allele (X² = 10.309, OR = 1.74; 95% CI = 1.24–2.45; *P* = 0.001324) were statistically significantly linked to an increased risk of developing BLCA. The CYP1A2-163 C/A polymorphism exhibited a significant association in both the dominant model (X² = 16.111, OR = 2.42; 95% CI = 1.57–3.75; *P* = 0.000060) and the recessive model (X² = 0.850, OR = 0.65; 95% CI = 0.26–1.63; *P* = 0.356) (Table 5). Additionally, BLCA patients exhibited a higher frequency of the CYP1A2-163 C/A CC genotype (0.38%) compared to controls (0.6%) (Table 6). These findings underscore the potential significance of specific genetic variants in the CYP1A2 gene in the etiology and progression of bladder cancer, suggesting that these polymorphisms may serve as genetic markers for assessing BLCA risk and warranting further investigation into their role in cancer development.

**Table 5:** Exact test for hardy-weinberg equilibrium of CYP1A2 SNPs

SNP	Groups	N11	N12	N22	N1	N2	*P* value
CYP1A2 -163 C.< A (rs762551)	All subjects	167	153	20	487	193	0.061
	BLC/A	65	93	12	223	117	0.0066
	Healthy_Control	102	60	8	264	76	1
CYP1A2.-3860.G<.A (rs2069514)	All subjects	195	127	18	517	163	0.77
	BLC/A	86	72	12	244	96	0.7
	Healthy_Control	109	55	6	273	67	1

OR (95% CI): Odds Ratio with corresponding 95% Confidence Interval. *P*-value: Statistical significance value

**Table 6:** Genotype and allele frequency distribution of CYP1A2-163 C/A

SNP	Genotype	BLC.A.(*n* = 170)	Healthy (*n* = 170)	X^2^	Odds (95% Cl)	*P*
CYP1A2 -163 C.< A (rs762551)	C/C	65(0.38)	102(0.6)	-	1.000 (ref.)	-
	C/A	93(0.55)	60(0.35)	12.941	2.21(1.43–3.42)	0.000321
	A/A	12(0.07)	8(0.05)	0.850	1.54(0.61–3.86)	0.356
	**Allele Frequencies**					
	C	223(0.66)	264(0.78)	--	--	--
	A	117(0.34)	76(0.22)	10.309	1.74(1.24–2.45)	0.001324
	**Genetic Model**					
Dominant	C/C	65 (38.2%)	102 (60%)	-	1.000 (ref.)	-
	C/A-A/A	105 (61.8%)	68 (40%)	16.111	2.42(1.57–3.75)	0.000060
Recessive	A/A	12 (7.1%)	8 (4.7%)	-	1.000 (ref.)	-
	C/C-C/A	158 (92.9%)	162 (95.3%)	0.850	0.65 (0.26–1.63)	0.356552
OVER DOMINANT	C/C-A/A	77 (45.3%)	110 (64.7%)	12.941	2.21(1.43–3.42)	0.000321
	C/A	93 (54.7%)	60 (35.3%)			

### Genotype and allele frequency distribution for CYP1A2-3860G/A

**Fig. 3:**
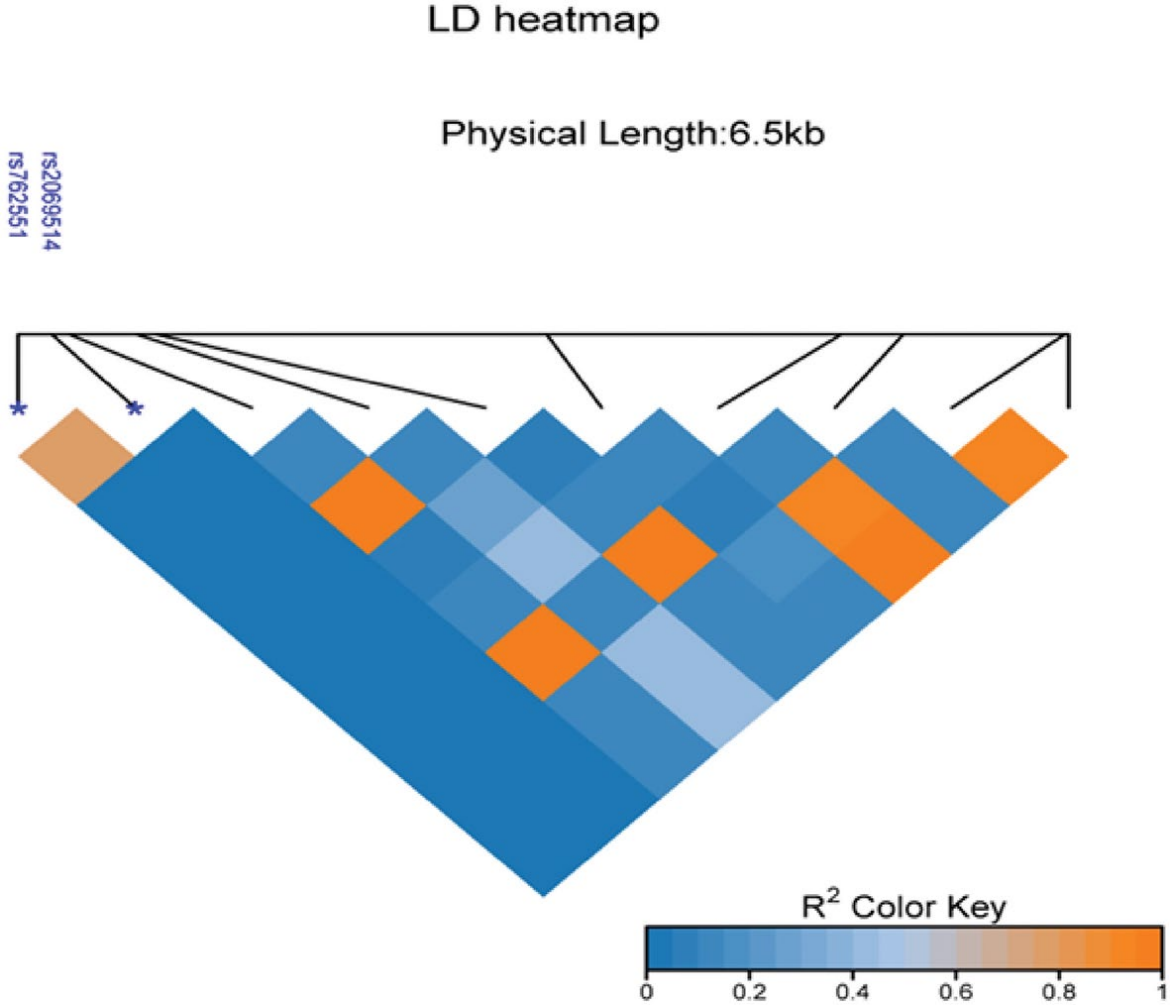
Linkage Disequilibrium (LD) heat map of CYP1A2 SNPs associated with rs762551 and rs2069514

In contrast to the healthy control group, patients with bladder cancer had considerably higher frequencies of the G allele (0.72%) and the GG genotype (0.57%) in rs2069514. However, as indicated in Table 7, the “A” allele frequency (0.24%) and AA genotype frequency (48%) in the patients were lower than the control group’s (0.32%) and (7.0%) rates, respectively. The − 3860G/A GA genotype (X2 = 3.632, OR = 1.54; 95% CI = 0.99–2.39; *P* = 0.056665) and G allele (X2 = 6.786, OR = 1.60; 95% CI = 1.12–2.29; *P* = 0.009187) were discovered to be statistically non-significantly linked to a higher risk of developing BLCA. The relationship of the CYP1A2-3860G/A polymorphism was non-significant (X2 = 2.112, OR = 2.08; 95% CI = 0.76–5.67; *P* = 0.146167) in both dominant models (G/G + G/A vs. A/A) and the recessive model (G/G vs. G/A + A/A). The findings are summarized in Table 7. The frequency of CYP1A2-3860G/A GG genotype (11.00%) in BLCA patients was higher than that of controls (7.0%) as shown in Table 7. The AA genotype exhibited a statistically significant association with an elevated risk of bladder cancer. However, the CC genotype was not significantly associated with bladder cancer risk (adjusted OR = 1.84; 95%CI = 0.793.28; *P* = 0.16). Therefore, in the research population, the “A” allele can be considered a risk allele, and the “C” allele is protective. Thus, it is proposed that in heterozygous C/A genotypes for rs762551, the A allele masks the C allele, probably as a result of complicated interactions when both alleles are co-dominant. In this case, it was theorized that the “C” allele triggers a protective reaction that shields a person from acquiring bladder cancer while the high frequency of the “A” allele may cause abnormal gene functioning or develop into cancer. The genotypes and alleles distribution of CYP1A2-163 C/A (rs762551) (Table 4). The LD heat map visually represents the extent of genetic linkage disequilibrium between CYP1A2 SNPs and the specific variants rs762551 and rs2069514 (Fig. 3). Darker colors or higher values indicate a stronger association, suggesting that these SNPs tend to be inherited together on the same chromosome. This analysis helps identify potential genetic relationships and co-inheritance patterns within the CYP1A2 genomic region (Fig. 3).

**Table 7:** Genotype and allele frequency of CYP1A2-3860 G/A (rs2069514) in Cancer and control groups

SNP	Genotype	BLC.A.(*n* = 170)	Healthy (*n* = 170)	X^2^	Odds (95% Cl)	*P*
CYP1A2.-3860.G<.A (rs2069514)	G/G	86(0.51)	109(0.64)	-	1.000 (ref.)	-
	G/A	72(0.42)	55(0.32)	3.632	1.54(0.99 − 0.239)	0.056665
	A/A	12(0.07)	6(0.04)	2.112	2.08(0.76–5.67)	0.146167
	**Allele Frequencies**					
	G	244(0.72)	273(0.8)	-	1.000 (ref.)	-
	A	96(0.28)	67(0.2)	**6.786**	1.60(1.12–2.29)	0.009187
	**Genetic Model**					
Dominant	G/G	86 (50.6%)	109 (64.1%)	-	1.000 (ref.)	-
	G/A-A/A	84 (49.4%)	61 (35.9%)	6.361	1.75(1.13–2.69)	0.011665
Recessive	G/G-G/A	158 (92.9%)	164 (96.5%)	2.112	2.08(0.18–1.36)	0.146167
	A/A	12 (7.1%)	6 (3.5%)	-	1.000 (ref.)	-
OVER DOMINANT	G/G-A/A	98 (57.6%)	115 (67.7%)	3.632	0.65(0.42–1.01)	0.056665
	C/A	72 (42.4%)	55 (32.4%)	-	1.000 (ref.)	-

### Haplotype frequencies and association analysis

The study examined the frequencies of haplotypes and their associations with the response variable within the study cohort. Haplotypes denote combinations of genetic variants inherited from a single parent. Four distinct haplotypes were examined based on the allelic combinations of rs762551 and rs2069514.

**Haplotype 1 (C/C)**: The first haplotype, characterized by the C/C genotype of the SNP CYP1A2.163 C.A, was observed in 65 individuals (38.2%) in the bladder cancer patients, while it was present in 102 individuals (60%) in the control group. In the codominant model, this haplotype yielded an AIC of 8 and a BIC of 23.3. In the dominant model (C/C), it was found in 65 individuals (38.2%) in the bladder cancer patients and 102 individuals (60%) in the control group, with AIC and BIC values of 6 and 17.5, respectively. In the recessive model (C/C-C/A), it was present in 158 individuals (92.9%) in the bladder cancer patients and 162 individuals (95.3%) in the control group, with AIC and BIC values of 6 and 17.5. Finally, in the over-dominant model (C/C-A/A), it was observed in 77 individuals (45.3%) in the bladder cancer patients and 110 individuals (64.7%) in the control group, with AIC and BIC values of 6 and j17.5 (Table 8).

**Table 8:** Haplotype analysis of CYP1A2.163 C.A and CYP1A2.3860.G.A bladder cancer

SNP	Model	Genotype	GROUP.BLC.A	GROUP.Healthy_Control	AIC	BIC
CYP1A2.163 C.A	Co-dominant	C/C	65 (38.2%)	102 (60%)	8	23.3
		C/A	93 (54.7%)	60 (35.3%)		
		A/A	12 (7.1%)	8 (4.7%)		
	Dominant	C/C	65 (38.2%)	102 (60%)	6	17.5
		C/A-A/A	105 (61.8%)	68 (40%)		
	Recessive	C/C-C/A	158 (92.9%)	162 (95.3%)	6	17.5
		A/A	12 (7.1%)	8 (4.7%)		
	Over- Dominant	C/C-A/A	77 (45.3%)	110 (64.7%)	6	17.5
		C/A	93 (54.7%)	60 (35.3%)		
CYP1A2.3860.G.A	Co-dominant	G/G	86 (50.6%)	109 (64.1%)	6	17.5
		G/A	72 (42.4%)	55 (32.4%)		
		A/A	12 (7.1%)	6 (3.5%)		
	Dominant	G/G	86 (50.6%)	109 (64.1%)	6	17.5
		G/A-A/A	84 (49.4%)	61 (35.9%)		
	Recessive	G/G-G/A	158 (92.9%)	164 (96.5%)	8	23.3
		A/A	12 (7.1%)	6 (3.5%)	6	17.5
	Over- Dominant	G/G-A/A	98 (57.6%)	115 (67.7%)	6	17.5
		C/A	72 (42.4%)	55 (32.4%)		

**Haplotype 2 (C/A)**: The second haplotype, characterized by the C/A genotype of the SNP CYP1A2.163 C.A, showed a prevalence of 93 individuals (54.7%) in the bladder cancer patients and 60 individuals (35.3%) in the control group. Notably, in the codominant model, no AIC or BIC values were provided. This haplotype did not appear to exhibit a distinct dominant or recessive pattern in the dataset (Table 8).

**Haplotype 3 (G/G)**: The third haplotype, defined by the G/G genotype of the SNP CYP1A2.3860.G.A, was found in 86 individuals (50.6%) in bladder cancer patients and 109 individuals (64.1%) in the control group in the codominant model. The AIC and BIC values were both 6 and 17.5, respectively. In the dominant model (G/G), this haplotype was observed in 86 individuals (50.6%) in the bladder cancer patients and 109 individuals (64.1%) in the control group, with AIC and BIC values of 6 and 17.5. In the recessive model (G/G-G/A), it was present in 158 individuals (92.9%) in the bladder cancer patients and 164 individuals (96.5%) in the control group, with AIC and BIC values of 8 and 23.3. For the over-dominant model (G/G-A/A), it was seen in 98 individuals (57.6%) in the bladder cancer patients and 115 individuals (67.7%) in the control group, with AIC and BIC values of 6 and 17.5 (Table 8).

**Haplotype 4 (G/A)**: The fourth haplotype, represented by the G/A genotype of the SNP CYP1A2.3860.G.A, was present in 72 individuals (42.4%) in the bladder cancer patients and 55 individuals (32.4%) in the control group in the codominant model. Unfortunately, AIC and BIC values were not provided for this haplotype. This haplotype did not appear to exhibit a distinct dominant or recessive pattern in the dataset (Table 8).

## Discussion

Bladder cancer’s (BLCA) etiology is still not fully understood. Genetic factors and many different pathways may have an impact on the development and progression of BLCA [21]. Researchers have discovered genetic polymorphisms (GPMs) in several genes that may be associated with BLCA. CYP1A2 plays a significant role in the metabolism of carcinogenic aromatic and heterocyclic amines, inhibiting this enzyme’s activity may be a straightforward way to stop the growth of malignancies in humans carried on by aromatic and heterocyclic amines. Genetic variations within the CYP1A2 gene are recognized to have multifaceted implications for cancer development [16]. Studies investigating the relationship between CYP1A2 polymorphisms and susceptibility to bladder cancer have yielded divergent results [22]. Elevated in vivo expression of CYP1A2 has been postulated as a risk factor for cancers affecting the bladder, colon, and rectum, where exposure to substances like aromatic amines and heterocyclic amines (HAs) has been implicated in the etiology of the disease [10, 11]. Furthermore, specific polymorphisms within the CYP1A2 gene have been demonstrated to influence CYP1A2 expression. Polymorphisms within the 5’ noncoding promoter region of the CYP1A2 gene [-3860G/A (rs2069514), -2467T/delT (rs3569413)], as well as intron 1 [-163 C/A (rs762551)], have been found to modify CYP1A2 expression in individuals who smoke [12].

Previous research has explored the association between CYP1A2 polymorphisms and the risk of bladder cancer, with varying outcomes [13]. Specifically, prior investigations have examined the connection between CYP1A2-163 C/A and − 3860 G/A polymorphisms and susceptibility to bladder carcinoma. Sun et al. conducted a meta-analysis to investigate the relationship between CYP1A2-163 C/A polymorphisms and cancer risk in various genetic models [23]. In the comprehensive meta-analysis of available data, a significant association between the CYP1A2-163 C/A polymorphism and cancer risk was discerned [24, 25]. In the present study, we focused on two single-nucleotide polymorphisms (SNPs) within the CYP1A2 gene, namely rs762551 and rs2069514. Our analysis revealed a noteworthy association between rs762551 and an elevated risk of bladder cancer (BLCA), while no statistically significant associations were observed between rs2069514 and BLCA susceptibility. These findings underscore a robust correlation between genetic variants of CYP1A2-163 C/A and the propensity for BLCA development. Notably, this study represents the first to establish a relationship between these SNPs in CYP1A2 and BLCA, based on a Pakistani cohort. It was observed that individuals diagnosed with bladder cancer exhibited significantly higher frequencies of the − 163 A allele compared to appropriately matched healthy control subjects. The frequencies of CC, CA, and AA genotypes were 36%, 45%, and 19% in BLCA patients, which significantly deviated from the frequencies in the control group (59.00%, 32%, and 9%, respectively). The AA genotype exhibited a robust association with an increased risk of bladder cancer. In contrast, for rs2069514, there were no discernible differences in genotype and allele frequencies between the BLCA and control groups. The frequencies of AA, GA, and GG genotypes were 48%, 41%, and 11% in BLCA patients, which did not significantly differ from the frequencies observed in the control group (59.00%, 34%, and 7%, respectively).

The outcomes of our investigation align with a study conducted in a Chinese population by Yong Zeng et al., which also found that the A allele of the CYP1A2 rs762551 (-163, C/A) SNP was linked to an increased risk of BLCA [26]. Our findings further corroborate the results reported by Wen-Xia Sun et al., who indicated a significant protective effect of the rs762551 CYP1A2 SNP homozygous mutant on bladder cancer within a Caucasian population [8, 19, 23]. Li Zhenzhen et al., in their examination of rs762551, noted that carriers of the C-allele had an elevated risk of cancer under one allele genetic model (C-allele vs. A-allele) but not under other models. Our subgroup analysis extended these findings and revealed a significant association under both dominant and recessive models. As for rs2069514, Li Zhenzhen et al. found no significant association with cancer risk across any genetic model (allele contrast, codominant, dominant, or recessive model), consistent with the observations of our present study [25].

## Conclusion

In conclusion, this investigation sheds light on a robust association between CYP1A2 gene polymorphisms, particularly the − 163 C/A allele, and an increased susceptibility to bladder cancer (BLCA). The meticulous odds ratio (OR) analysis underscores the significance of the rs762551 (CYP1A2-163 C/A) and rs2069514 (CYP1A2-3860G/A) variants, positioning them as potential genetic markers for heightened BLCA risk. The C/A genotype of rs762551 displays a notable prevalence in BLCA cases, accompanied by an OR of 2.21, while the A/A genotype suggests an increased risk, though statistical significance eludes. Correspondingly, the rs2069514 G/A genotype implies susceptibility to BLCA, and the A/A genotype hints at a potential correlation. Haplotype analysis further refines comprehension, unveiling distinct combinations, with particular prominence accorded to the C-G haplotype. These revelations furnish invaluable insights into the genetic underpinnings of BLCA, laying a sturdy groundwork for personalized therapeutic strategies. Nevertheless, the imperative for extended research across diverse populations persists, ensuring the robust validation of these associations and the refinement of translating genetic insights into effective clinical strategies for managing bladder cancer.

### Electronic supplementary material

Below is the link to the electronic supplementary material.

Supplementary Material 1

## Data Availability

No datasets were generated or analysed during the current study.

## References

[1] Murta-Nascimento C, Schmitz-Dräger BJ, Zeegers MP, Steineck G, Kogevinas M, Real FX et al. Epidemiology of urinary bladder cancer: from tumor development to patient’s death. World J Urol [Internet]. 2007;25(3):285–95. 10.1007/s00345-007-0168-5. 10.1007/s00345-007-0168-5 17530260

[2] Elsalem L, Alfaqih MA, Al Bashir S, Halalsheh O, Basheer HA, Mhedat K et al. Genetic variation in the ADIPOQ gene and serum adiponectin increase the risk of bladder cancer. J Appl Biomed [Internet]. 2022;20(3):106–13. 10.32725/jab.2022.012. 10.32725/jab.2022.012 36218131

[3] Siegel R, Naishadham D, Jemal A. Cancer statistics for Hispanics/Latinos, 2012. CA Cancer J Clin [Internet]. 2012;62(5):283–98. 10.3322/caac.21153. 10.3322/caac.21153 22987332

[4] Berdik C. Unlocking bladder cancer. Nature [Internet]. 2017;551(7679):S34–5. 10.1038/551s34a. 10.1038/551S34a 29117159

[5] Inamura K. Bladder Cancer: New Insights into Its Molecular Pathology. Cancers (Basel) [Internet]. 2018;10(4):100. https://pubmed.ncbi.nlm.nih.gov/29614760. 10.3390/cancers10040100 PMC5923355 29614760

[6] Janisch F, Shariat SF, Schernhammer E, Rink M, Fajkovic H. The interaction of gender and smoking on bladder cancer risks. Curr Opin Urol [Internet]. 2019;29(3):249–55. 10.1097/mou.0000000000000602. 10.1097/MOU.0000000000000602 30888973

[7] Ashrafizadeh M, Zarrabi A, Karimi-Maleh H, Taheriazam A, Mirzaei S, Hashemi M et al. Author response for (Nano)platforms in bladder cancer therapy: Challenges and opportunities [Internet]. Wiley; 2022. 10.1002/btm2.10353/v2/response1. 10.1002/btm2.10353 PMC9842064 36684065

[8] Vukovic V, Ianuale C, Leoncini E, Pastorino R, Gualano MR, Amore R et al. Lack of association between polymorphisms in the CYP1A2 gene and risk of cancer: evidence from meta-analyses. BMC Cancer [Internet]. 2016;16:83. https://pubmed.ncbi.nlm.nih.gov/26865042. 10.1186/s12885-016-2096-5 PMC4750358 26865042

[9] Tao L, Xiang YB, Chan KK, Wang R, Gao YT, Yu MC et al. Cytochrome P4501A2 phenotype and bladder cancer risk: The Shanghai bladder cancer study. Int J Cancer [Internet]. 2011/06/21. 2012;130(5):1174–83. https://pubmed.ncbi.nlm.nih.gov/21480221. 10.1002/ijc.26121 PMC3167995 21480221

[10] Datta N, Chakraborty S, Basu M, Ghosh MK. Tumor Suppressors Having Oncogenic Functions: The Double Agents. Cells [Internet]. 2020;10(1):46. https://pubmed.ncbi.nlm.nih.gov/33396222. 10.3390/cells10010046 PMC7824251 33396222

[11] Tripathi A, Kashyap A, Tripathi G, Yadav J, Bibban R, Aggarwal N et al. Tumor reversion: a dream or a reality. Biomark Res [Internet]. 2021;9(1):31. https://pubmed.ncbi.nlm.nih.gov/33958005. 10.1186/s40364-021-00280-1 PMC8101112 33958005

[12] Gunes A, Ozbey G, Vural EH, Uluoglu C, Scordo MG, Zengil H et al. Influence of genetic polymorphisms, smoking, gender and age on CYP1A2 activity in a Turkish population. Pharmacogenomics [Internet]. 2009;10(5):769–78. 10.2217/pgs.09.22. 10.2217/pgs.09.22 19450128

[13] Yin X, Xiong W, Wang Y, Tang W, Xi W, Qian S et al. Association of CYP2E1 gene polymorphisms with bladder cancer risk: A systematic review and meta-analysis. Medicine (Baltimore) [Internet]. 2018;97(39):e11910–e11910. https://pubmed.ncbi.nlm.nih.gov/30278485. 10.1097/MD.0000000000011910 PMC6181476 30278485

[14] Chevalier D, Cauffiez C, Allorge D, Lo-Guidice JM, Lhermitte M, Lafitte JJ, et al. Five novel natural allelic variants?951A&gt;C, 1042G&gt;A (D348N), 1156A&gt;T (I386F), 1217G&gt;A (C406Y) and 1291C&gt;T (C431Y)?of the human CYP1A2 gene in a French Caucasian population. Hum Mutat [Internet]. 2001;17(4):355–6. Available from: 10.1002/humu.49 11295848

[15] Sankhwar M, Sankhwar SN, Bansal SK, Gupta G, Rajender S. Polymorphisms in the XPC gene affect urinary bladder cancer risk: a case-control study, meta-analyses and trial sequential analyses. Sci Rep [Internet]. 2016;6:27018. https://pubmed.ncbi.nlm.nih.gov/27246180. 10.1038/srep27018 PMC4887911 27246180

[16] Pavanello S, Mastrangelo G, Placidi D, Campagna M, Pulliero A, Carta A et al. CYP1A2 polymorphisms, occupational and environmental exposures and risk of bladder cancer. Eur J Epidemiol [Internet]. 2010;25(7):491–500. 10.1007/s10654-010-9479-8. 10.1007/s10654-010-9479-8 20559687

[17] Nebert DW, Dalton TP. The role of cytochrome P450 enzymes in endogenous signalling pathways and environmental carcinogenesis. Nat Rev Cancer [Internet]. 2006;6(12):947–60. 10.1038/nrc2015. 10.1038/nrc2015 17128211

[18] Vilčková M, Škereňová M, Dobrota D, Kaplán P, Jurečeková J, Kliment J et al. Polymorphisms in the gene encoding CYP1A2 influence prostate cancer risk and progression. Oncol Lett [Internet]. 2023;25(2):85. https://pubmed.ncbi.nlm.nih.gov/36760517. 10.3892/ol.2023.13671 PMC9878356 36760517

[19] Dobrinas M, Cornuz J, Oneda B, Kohler Serra M, Puhl M, Eap CB. Impact of Smoking, Smoking Cessation, and Genetic Polymorphisms on CYP1A2 Activity and Inducibility. Clin Pharmacol & Ther [Internet]. 2011;90(1):117–25. 10.1038/clpt.2011.70. 10.1038/clpt.2011.70 21593735

[20] Guengerich FP, Parikh A, Turesky RJ, Josephy PD. Inter-individual differences in the metabolism of environmental toxicants: cytochrome P450 1A2 as a prototype. Mutat Res Mol Mech Mutagen [Internet]. 1999;428(1–2):115–24. 10.1016/s1383-5742(99)00039-3. 10.1016/s1383-5742(99)00039-3 10517985

[21] Zeng Y, Jiang HY, Wei L, Xu WD, Wang YJ, Wang YD et al. Association between the CYP1A2 rs762551 Polymorphism and Bladder Cancer Susceptibility: a Meta-Analysis Based on Case-Control Studies. Asian Pacific J Cancer Prev [Internet]. 2015;16(16):7249–54. 10.7314/apjcp.2015.16.16.7249. 10.7314/apjcp.2015.16.16.7249 26514519

[22] Murakami K, Furuya H, Hokutan K, Goodison S, Pagano I, Chen R et al. Association of SNPs in the PAI1 Gene with Disease Recurrence and Clinical Outcome in Bladder Cancer. Int J Mol Sci [Internet]. 2023;24(5):4943. https://pubmed.ncbi.nlm.nih.gov/36902377. 10.3390/ijms24054943 PMC10003630 36902377

[23] Sun WX, Chen YH, Liu ZZ, Xie JJ, Wang W, Du YP et al. Association between the CYP1A2 polymorphisms and risk of cancer: a meta-analysis. Mol Genet Genomics [Internet]. 2014;290(2):709–25. 10.1007/s00438-014-0956-8. 10.1007/s00438-014-0956-8 25472037

[24] Pavanello S, Pulliero A, Lupi S, Gregorio P, Clonfero E. Influence of the genetic polymorphism in the 5′-noncoding region of the CYP1A2 gene on CYP1A2 phenotype and urinary mutagenicity in smokers. Mutat Res Toxicol Environ Mutagen [Internet]. 2005;587(1–2):59–66. 10.1016/j.mrgentox.2005.08.008. 10.1016/j.mrgentox.2005.08.008 16188490

[25] Zhenzhen L, Xianghua L, Ning S, Zhan G, Chuanchuan R, Jie L. Current evidence on the relationship between three polymorphisms in the CYP1A2 gene and the risk of cancer. Eur J Cancer Prev [Internet]. 2013;22(6):607–19. 10.1097/cej.0b013e32835f3bd2. 10.1097/CEJ.0b013e32835f3bd2 23462460

[26] Ghotbi R, Christensen M, Roh HK, Ingelman-Sundberg M, Aklillu E, Bertilsson L. Comparisons of CYP1A2 genetic polymorphisms, enzyme activity and the genotype-phenotype relationship in Swedes and Koreans. Eur J Clin Pharmacol [Internet]. 2007;63(6):537–46. 10.1007/s00228-007-0288-2. 10.1007/s00228-007-0288-2 17370067

