# Supplementary material for: Association between CYP1A2 gene variants −163 C/A (rs762551) and −3860 G/A (rs2069514) and bladder cancer susceptibility
Source: BMC Cancer. 2024 Jul 22;24:880. doi: 10.1186/s12885-024-12553-7 (PMC11262005; doi:10.1186/s12885-024-12553-7)
Supplement: Supplementary file 1 — Supplementary Material 1 [file 12885_2024_12553_MOESM1_ESM.docx]

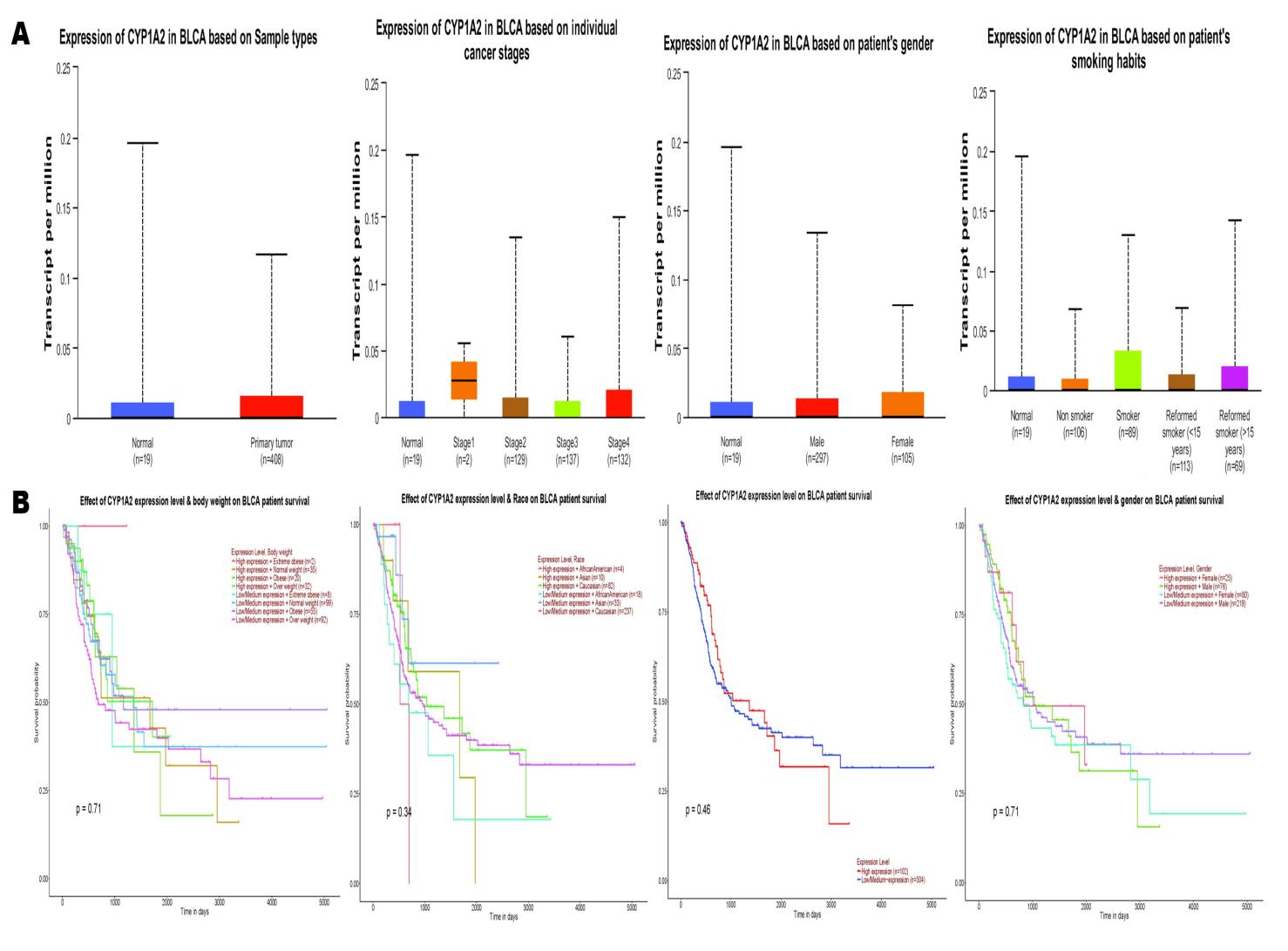


**Figure S1: CYP1A2 gene Survival analysis on TCGA samples of bladder cancer in smokers, non-smokers, gender in different stages**


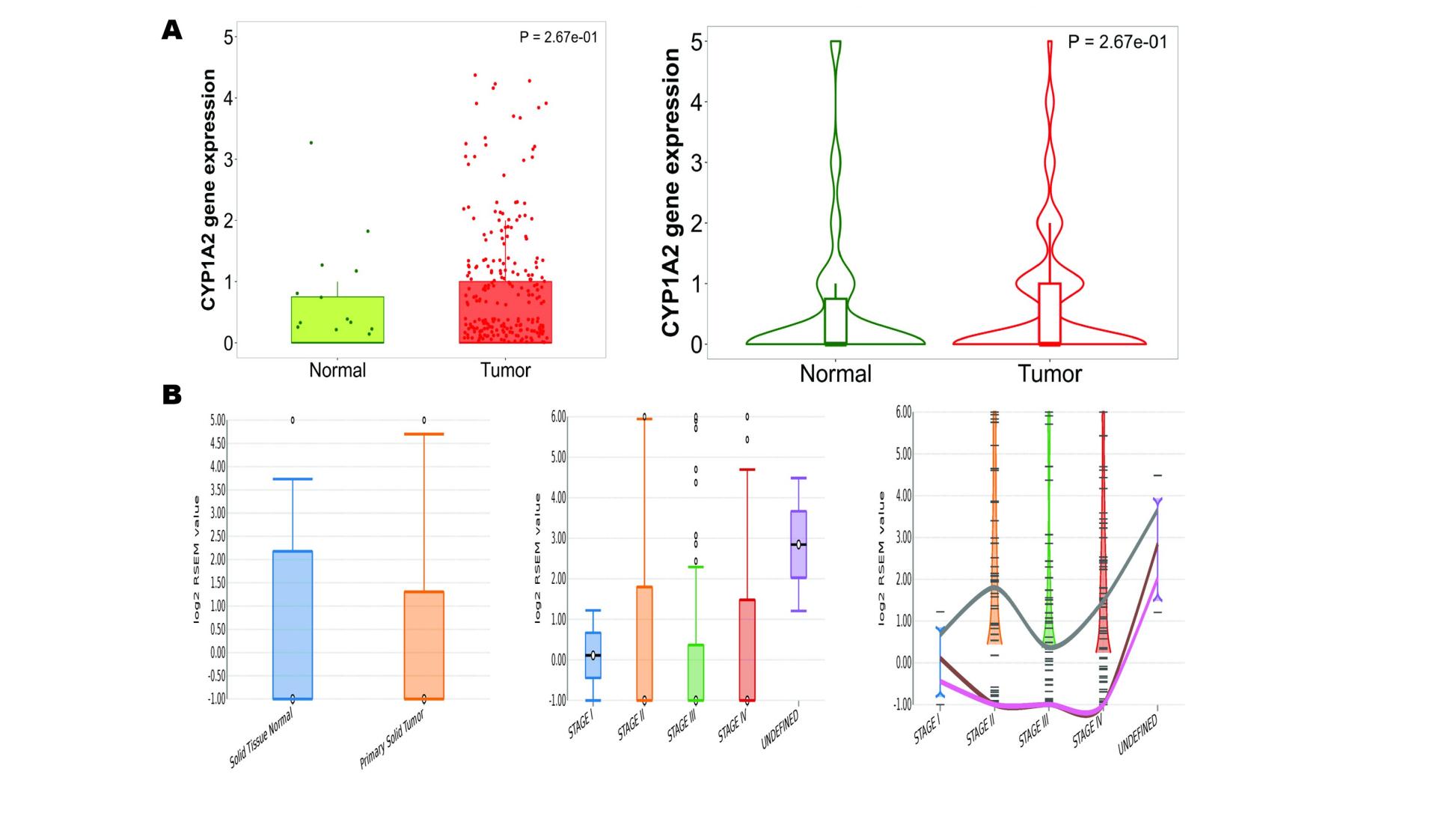


**Figure S2.** Expression Analysis of CYP1A2 gene on TCGA samples of bladder cancer using ULCAN database
